# Stability constraints on large-scale structural brain networks

**DOI:** 10.3389/fncom.2013.00031

**Published:** 2013-04-12

**Authors:** Richard T. Gray, Peter A. Robinson

**Affiliations:** ^1^The Kirby Institute, The University of New South WalesSydney, NSW, Australia; ^2^School of Physics, University of SydneySydney, NSW, Australia; ^3^Brain Dynamics Center, Sydney Medical School – Western, University of SydneyWestmead, NSW, Australia

**Keywords:** brain networks, stability, network spectra, random matrices, mean-field modeling

## Abstract

Stability is an important dynamical property of complex systems and underpins a broad range of coherent self-organized behavior. Based on evidence that some neurological disorders correspond to linear instabilities, we hypothesize that stability constrains the brain's electrical activity and influences its structure and physiology. Using a physiologically-based model of brain electrical activity, we investigated the stability and dispersion solutions of networks of neuronal populations with propagation time delays and dendritic time constants. We find that stability is determined by the spectrum of the network's matrix of connection strengths and is independent of the temporal damping rate of axonal propagation with stability restricting the spectrum to a region in the complex plane. Time delays and dendritic time constants modify the shape of this region but it always contains the unit disk. Instabilities resulting from changes in connection strength initially have frequencies less than a critical frequency. For physiologically plausible parameter values based on the corticothalamic system, this critical frequency is approximately 10 Hz. For excitatory networks and networks with randomly distributed excitatory and inhibitory connections, time delays and non-zero dendritic time constants have no impact on network stability but do effect dispersion frequencies. Random networks with both excitatory and inhibitory connections can have multiple marginally stable modes at low delta frequencies.

## Introduction

The brain is possibly the most complicated example of a system of interacting dynamical units whose activity self-organizes to produce complex global behavior. The human brain performs cognitive functions through the transmission of action potentials within a vast structurally dynamic network consisting of approximately 10^11^ neurons and up to 10^15^ synaptic interconnections (Kandel et al., 2000; Koch, 2004; Sporns et al., 2005). The aggregate of all neural firings within this network results in large-scale coherent electrical activity and the performance of high-level cognitive functions. Understanding the structure and physiology of the brain thus gives insight into its overall behavior.

At large scales the excitatory and inhibitory neurons in the brain are organized into a complex large-scale network of distinct anatomical and functional structures (Sporns et al., 2004, 2005; Bullmore and Sporns, 2009; van den Heuvel and Sporns, 2011) We can represent this structure as a complex network—the structure of which has been studied extensively in recent times with a number of experimental cortical connection networks determined for the cat and the macaque monkey (Felleman and van Essen, 1991; Scannell et al., 1995; Jouve et al., 1998; Hilgetag et al., 2000a,b; Sporns, 2011). These networks have a modular hierarchical structure with the small-world properties of high local clustering and short path length between structures (Hilgetag et al., 2000a,b; Sporns et al., 2000, 2004, 2005; Young, 2000; Sporns and Zwi, 2004; Bassett and Bullmore, 2006; Bullmore and Sporns, 2009, 2012).

Reasons for why the brain has evolved this particular large-scale structure are currently unknown. A number of investigations have concentrated on the effect of physical constraints on brain structure. Such constraints include brain volume, wiring length, and energy consumption or metabolic demands (Laughlin et al., 1998; Attwell and Laughlin, 2001; Lennie, 2003). Other studies have looked at functional constraints such as minimizing the conduction delay or processing steps for a signal to travel from one neuron to another (Wen and Chklovskii, 2005). Alternatively, the dynamics of the brain's electrical activity may constrain the brain's structure. If the physiology and structural characteristics of the brain produce adverse electrical activity resulting in seizures, tremors, or other neurological disorders then it is likely the structural characteristics of the brain will be constrained to limit these disorders.

One of the most important dynamical properties of complex systems such as the brain is stability. It has been associated with pattern formation (Turing, 1952; Murray, 2002), synchronized activity (Kuramoto, 1984; Pecora and Carroll, 1998; Jirsa and Ding, 2004; Acebrón et al., 2005; Feng et al., 2006), the complexity and diversity of ecosystems (May, 1972, 1974; Hogg et al., 1989; McCann, 2000; Allesina and Tang, 2012), the functioning of biological systems (Murray, 2002; Taverna and Goldstein, 2002; Steuer, 2007), and the generation of coherent self-organized behavior. Stability is also an important aspect of the design and control of advanced technological systems (Bechhoefer, 2005).

A common approach to studying the large-scale dynamics of the brain's electrical activity is to use a continuum mean-field approximation for neural activity. This approach has been extensively studied over the past 30 years producing numerous models for the electrical activity within the brain (Wilson and Cowan, 1973; da Silva et al., 1974; Nunez, 1974, 1995; Freeman, 1975; Steriade et al., 1990; Jirsa and Haken, 1996; Wright and Liley, 1996; Robinson et al., 1997, 2003a; Wright et al., 2001; Robinson, 2005). This work has been reviewed recently (Deco et al., 2008; Bressloff, 2011). These models have been used extensively to perform stability analysis and understand pattern formation, oscillations and waves in the brain's electrical activity (Deco et al., 2008; Bressloff, 2011).

Measurements of brain activity suggest that the brain operates close to marginal stability, permitting a wide range of flexible, adaptable, and complex behavior (Stam et al., 1999; Robinson et al., 2001b; Breakspear, 2002; Breakspear et al., 2003). Physiological modeling also suggests that linear instabilities in the brain's electrical activity correspond to neurological disorders, such as epilepsy (Robinson et al., 1998, 2002; Breakspear et al., 2006; Kim and Robinson, 2007; Deco et al., 2008). It is therefore possible that stability is a dynamical property that imposes constraints on the brain's physiology and structure.

In previous work we have used a simplified version of the Robinson, Rennie, Wright (RRW) physiologically-based continuum (mean-field) model (Robinson et al., 1997, 1998, 2003a; Wright et al., 2001; Rennie et al., 2002; Robinson, 2003, 2005) to study the dynamics of the electrical activity in large-scale structural brain networks. Based on the hypothesis that stability is a dynamical constraint on the structure and physiology of structural brain networks we have investigated the effect of stability of large-scale structural brain networks (Gray and Robinson, 2006, 2008, 2009b,a; Robinson et al., 2009).

Our previous work ignored the dendritic time constants of neurons and the propagation time delays for signals to travel between neural populations. However, time delays due to axonal propagation affect stability and the possible physiology of neuronal networks (Atay and Hutt, 2004; Jirsa and Ding, 2004; Coombes, 2005; Coombes et al., 2007; Qubbaj and Jirsa, 2007, 2009; Venkov et al., 2007; Jirsa, 2009). This previous work has generally used integro-differential neural field equations with connectivity within a neural mass described by homogeneous or heterogeneous kernels. Our approach here is to focus on the temporal dynamics of the overall electrical activity of arbitrarily connected large-scale structural brain networks, ignoring the spatial spread and propagation of electrical activity within individual neuronal populations.

In this study, we increase the physiological realism of our structural brain network model by allowing propagation time delays and non-zero dendritic time constants. After reviewing the stability of structural brain networks, we aim to investigate how these physiological features might affect the dynamics and stability of networks of neuronal populations where the connection patterns between populations are arbitrary—ignoring the spatial and geometric placement of the populations and simply focusing on which populations are inter-connected.

We also investigate the dispersion frequencies of marginally stable modes of electrical activity using plausible physiological parameters. The incorporation of non-zero dendritic time constants generalizes the work in (Jirsa and Ding, 2004) which, by including time delays, extended May's original analysis on the stability of complex systems (May, 1972, 1974).

## Methods

A structural brain network of *n* neural populations is represented by a directed graph *N* whose vertices and edges represent specific neural populations and inter-population connections, respectively. Neural populations within a network are collections of neurons with an assumed effective range and of sufficient number for a mean-field approximation to be valid. For example, a neural population can represent all the neurons in a distinct region or nuclei of the brain (e.g., cortical area, thalamus), a particular neuron type (e.g., interneuron, pyramidal cell), or a particular neurotransmitter type (e.g., glutamate, GABA, dopamine). Neurons in one population do not have to be separated geometrically or physically within the brain and can be intermixed with the neurons of another population (e.g., excitatory and inhibitory neurons in the cortex).

The structure of *N* is represented by a connection matrix **C**(*N*) = [*C*_*ab*_]; where *C*_*ab*_ = 1 if there is a connection from population *b* to population *a, C*_*ab*_ = 0 otherwise. If *C*_*ab*_ = *C*_*ba*_ for all *a* and *b*, the network is symmetric; otherwise it is asymmetric. Self-connections in structural brain networks correspond to non-zero diagonal entries in **C**(*N*). The connection matrix simply records whether one neuronal population sends neural signals to another neuronal population. Properties of connections are not included in **C**(*N*).

### Physiologically-based structural brain network dynamics

In this section we outline the physiological model used to describe the dynamics of a brain network. If a neural population contains a sufficient number of neurons a continuum approximation can be used, whereby the properties of population neurons are averaged over. This approximation is valid for length scales greater than a few tenths of a millimeter and is thus suitable for investigating the dynamics of large-scale structural brain networks.

The continuum approximation allows the use of a previously developed model, the RRW model, for the brain's electrical activity (Robinson et al., 1997; Wright et al., 2001; Rennie et al., 2002; Robinson et al., 2003b, 2004; Robinson, 2005). This continuum model incorporates and describes three features of neural dynamics: (1) the synapto-dendritic dynamics resulting in the cell body potential; (2) from the mean cell body potential an average firing rate is determined via a non-linear sigmoid function; and (3) the population firing rate generates a neural pulse forming a field ϕ(*t*) that propagates along the populations outgoing connections. The field within a population is temporally described using a damped wave equation. Implicitly the neurons in each population are assumed to have an effective range which gives a rate at which spikes reach axonal terminals and cease existance.

This model has been extensively used to model the corticothalamic system with the linear version having been shown to produce excellent agreement with EEG spectra, ERP, and other neurophysical phenomena (Robinson et al., 1997, 2001a,b; Rennie et al., 2002; Robinson, 2003). To apply this continuum model to brain networks we previously used a number of simplifying assumptions. In particular, we assumed that all neural populations have instantaneous dendritic response times and there is no time delay for a signal to be sent from one population to the other (Gray and Robinson, 2006, 2008, 2009b,a; Robinson et al., 2009). For this study we relax some of these assumptions.

Firstly we assume time delays τ for a signal to be sent from one population to another are equal. Secondly, we assume each population in a network has the same dendritic decay rate α and rise rate β. The values of 1/α and 1/β equal the dendritic decay and rise time constants, respectively; instantaneous rise and decay times imply 1/α = 1/β = 0. These assumptions are unrealistic for real structural brain networks but improve our previous analysis and allow us to analytically determine stability. Though for some structural brain networks these assumptions may be good approximations of the networks physiology. Another weakness of these assumptions is self-connections have the same time delay as connections between distinct populations—if τ ≠ 0 then self-connections in a network also involve a delay. Generally, self-connections represent interconnections within a neural population and would be expected to have zero time delay. However, for cortical networks self-connections can be used to represent feedback from underlying structures such as the thalamus. Time delayed self-connections would be appropriate for this type of feedback. We will generalize the assumption of equal time delays in future work.

The neurophysics and neurophysiology incorporated into the general RRW model and the equations for the linear perturbations of the neural field ϕ_*a*_, for each neural population *a*, are described and derived in detail elsewhere (Robinson et al., 1997; Wright et al., 2001; Rennie et al., 2002; Robinson et al., 2003b, 2004; Robinson, 2005). This study uses the notation and equations derived in (Robinson, 2005). Under the assumptions used here the RRW equations describing linear perturbations of the neural field ϕ_*a*_ of population *a* about the assumed steady state in Fourier space reduce to

(1) $${\left(1-i\omega /\gamma \right)}^{2}\phi_{a}(\omega )=\sum_{b}L(\omega )G_{ab}e^{i\omega \tau }\phi_{b}(\omega ),$$

(2) $$=L(\omega )e^{i\omega \tau }\sum_{b}G_{ab}\phi_{b}(\omega ),$$

where ω is the angular frequency and

(3) $$L(\omega )=\frac{\alpha \beta }{\left(\alpha -i\omega )(\beta -i\omega \right)}=\frac{1}{\left(1-i\omega /\alpha )(1-i\omega /\beta \right)}.$$

The gain *G*_*ab*_ is a dimensionless quantity describing the effect of changes in the firing rate of neurons in population *b* on the neurons of population *a*. Physiologically, *G*_*ab*_ is the number of extra action potentials produced in *a* per extra action potential incident from *b*. Hence, *G*_*ab*_ is a measure of how sensitive and responsive *a* is to changes in *b*'s activity. In the general RRW model γ is a damping rate equal to the velocity of the ϕ's propagation within a neural population divided by the characteristic range of the axons that carry it. In the spatially uniform case used here, γ represents a temporal damping rate.

Letting **G** = [*G*_*ab*_] be the matrix of gains and setting

(4) $$D(\omega )={\left[L\left(\omega \right)\right]}^{-1}\left(1-i\omega /\gamma \right)e^{-i\omega \tau },$$

(5) $$=(1-i\omega /\alpha )(1-i\omega /\beta ){\left(1-i\omega /\gamma \right)}^{2}e^{-i\omega \tau },$$

which is a complex analytic function. Equation (1) can be written in matrix form as

(6) Δ(ω)Φ(ω)=GΦ(ω),

where **Φ** is a column vector of the ϕ_*a*_ and **Δ**(ω) = *D*(ω)**I**, where **I** is the identity matrix. Setting **A** = **G** − **Δ**, Equation (6) can be simplified to

(7) A(ω)Φ(ω)=0.

The linear stability of a network is then determined by the solutions ω of the dispersion relation,

(8) det[A(ω)]=0.

The gain matrix **G** = [*G*_*ab*_] encodes all of the information in **C**, since *G*_*ab*_ ≠ 0 implies *C*_*ab*_ ≠ 0, as well as the strength of connections between populations. No assumptions (such as homogeneity or isotropy) are made for the characteristics of a connection and any attenuation or phase shifting of an incoming signal due to time delays are reflected in the exponential term of Equation (1). However, the model implicitly assumes an effective range for neurons within a population. If *G*_*ab*_ > 0 then the connection is excitatory and if *G*_*ab*_ < 0 the connection is inhibitory. Note that Equation (5) shows that if the values of α and β are exchanged, the brain network has the same dynamics and stability.

### Realistic parameter values for large-scale structural brain networks

Physiologically plausible parameter values for γ, α, β, and τ are shown in Table 1. These values are based on the parameters used in the corticothalamic model (Robinson et al., 2003a, 2004), with the specific values taken from (Robinson et al., 2004). The nominal values in Table 1 are the default model parameters used to illustrate our results under our assumptions α and β are the same value for all populations and τ is the same for all connections. The values for γ are based on the cortical excitatory neurons which form the long range connections within the cortex. Inhibitory inter-neurons in the cortex are short range (Nunez, 1995) and therefore have γ ≈ ∞. Under the assumptions used here all neural populations are given the same γ value.

**Table 1:** **Physiologically plausible ranges and nominal values of parameters**.

**Parameter**	**Range**	**Nominal value**	**Unit**
γ	30–220	100	s^−1^
α	5–200	60	s^−1^
β	17–2500	240	s^−1^
β/α	1–10	4	–
τ	0–50	10	ms

Based on the corticothalamic model parameters in Robinson et al. (2004). The nominal values are the default model parameters we use in this work.

In real structural brain networks dendritic time constants and propagation time delays may vary. The spatial distribution and physical separation of structures within the brain will lead to distinct time delays. The values of τ in Table 1 are physiologically plausible values for the time delays based on corticothalamic modeling. The nominal value is a realistic value for the average delay between the large-scale neural populations (or areas) in the cerebral cortex.

### Randomly connected large-scale structural brain networks

To illustrate our results we investigate randomly connected structural brain networks where neural populations are connected randomly with probability *p*. The size *n* and probability of connection we use is based on experimentally determined cortical connection networks for animals. These have been analyzed with graph-theoretical methods and all of these networks have less than 100 neural populations with a connection density (percentage of existing connections out all possible connections) of 20–40% (Felleman and van Essen, 1991; Scannell et al., 1995; Hilgetag et al., 2000a; Sporns et al., 2000, 2004; Bullmore and Sporns, 2009; Rubinov and Sporns, 2010; Sporns, 2011). We use random networks with *n* = 50 and *p* = 0.5 to illustrate our results, allowing comparisons with real cortical networks. These values of *p* ensure the networks are strongly connected (Bollobás, 1985) and all populations have at least one input and one output with high probability; i.e., there are no sources or sinks of electrical activity.

The specific random networks we investigate are the same random networks we have previously investigated (Gray and Robinson, 2006, 2008, 2009a,b). These networks consist of excitatory and inhibitory connections. The probability that a connection is inhibitory is given by *p*_*i*_ and such a connection has a negative gain. Excitatory gains are given values from a normal distribution with a mean μ_*e*_ > 0 and variance σ^2^_*e*_. Similarly, inhibitory connections have a gain sampled from a normal distribution with μ_*i*_ < 0 and variance σ^2^_*i*_. In terms of the gain matrix **G** all positive entries are sampled from 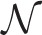
(μ_*e*_, σ^2^_*e*_) and all negative entries are sampled from 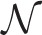
(μ_*i*_, σ^2^_*i*_).

Based on these parameters we investigated the stability of three types of networks: random networks with fixed excitatory gains (RENs), random connection networks (RCNs) with excitatory and inhibitory connections distributed randomly within the network (Gray and Robinson, 2009b), and random population networks (RPNs) (Gray and Robinson, 2009a). RPNs represent random networks with excitatory and inhibitory populations of neurons, this implies the outgoing connections of a given populations are all excitatory or all inhibitory. The gain matrix of RPNs consists of columns with either all entries ≤ 0 or all entries ≥ 0.

We determine the dispersion solutions for these structural brain networks numerically using a FORTRAN program called CROOT (Botten et al., 1983). This program finds dispersion solutions by implementing a recursive algorithm that employs Cauchy's integral formula (Mitrinović and Keckić, 1984) within a specified annulus or disk.

## Results

Our results describe the stability of structural brain networks by determining the criteria for a network to stable—starting from simple excitatory networks and then adding time delays and dendritic time constants. For our network model we show that stability is determined by the eigenvalues of the gain matrix with stability constraining the eigenvalues to a specific zone in the complex plain. The first subsections translate the results from previous work into the current context. In particular, when time delays are included we produce a similar tear-drop shaped stability zone found by others (Jirsa and Ding, 2004; Feng et al., 2006; Qubbaj and Jirsa, 2007; Jirsa, 2009; Qubbaj and Jirsa, 2009). However, we show the addition of dendritic time constants modifies the shape of the stability zone. Finally, we use our results to assess how stability constrains the physiology of randomly connected networks with excitatory and inhibitory connections.

### Stability of structural brain networks

The solutions ω of the dispersion relation Equation (8) determine the linear stability of a network. Setting λ = *D*(ω), the dispersion relation is

(9) det(G−λI)=0.

Therefore, network stability is determined by the spectrum of **G**, which we denote Sp(**G**). All the dispersion solutions ω of the network can be obtained by solving

(10) λ−D(ω)=0

for each λ in Sp(**G**). If all the λ in Sp(**G**) have corresponding ω [given by Equation (10)] with Imω < 0 then the network is stable. However, if there exists one λ which has a corresponding dispersion solution with Imω ≥ 0 then the network is unstable. The set of dispersion solutions of a brain network is termed the *dispersion spectrum*. Taking the complex conjugate of Equations (4, 10) show that if ω_1_ = Reω + *i*Imω = ω_*r*_ + *i*ω_*i*_ is solution for λ then ω_2_ = −ω_*r*_ + *i*ω_*i*_ is a solution for the complex conjugate $\bar{\lambda }$ of λ. Therefore, since both λ and $\bar{\lambda }$ are in Sp(**G**), the dispersion spectrum is symmetric about the real axis.

Solving Equation (10) for ω is equivalent to solving λ −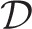
(ϖ) = 0 for ϖ where 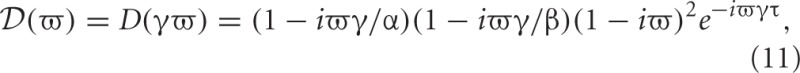 and ϖ = ω/γ is a dimensionless frequency parameter. From the ϖ solutions the dispersion solutions for the network are ω = γϖ. Since *D* and 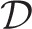 are analytic, the dispersion spectrum can be obtained by numerically solving Equation (10) for each λ.

#### Boundary between unstable and stable states

As the stability of a network is determined by Sp(**G**) we are interested in the zone of the complex plane where the all the eigenvalues of **G** must lie for the network to be stable. If a dispersion solution has Imω = 0 (i.e., ω is real and marginally stable) then the λ corresponding to ω lies on the critical boundary between the unstable and stable zones in the complex plane. Therefore, the stability boundary is given by *D*(ω) for real ω ranging from −∞ to ∞. To describe the stability boundary we use 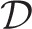
(ϖ) with real ϖ. This function traces out a continuous curve in the complex plane as ϖ ranges from −∞ to ∞. Points on this curve represent λ in Sp(**G**) with real dispersion solutions ω = γϖ. The stability boundary of a network is a segment of this curve since, in general, 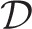
(ϖ) can intersect itself to form loops.

Since 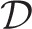 is a re-parameterization of *D* using ϖ = ω/γ, a brain network with model parameters γ = γ′, α = α′, β = β′, and τ = τ' has the same stability boundary as a network with γ = 1, α = α′/γ′, β = β′/γ′, and τ = γ′τ. However, the dispersion spectra of these networks will differ. If an initially stable network becomes unstable due to changes in its connection gains, its spectrum initially lies in the stability zone before at least one eigenvalue moves across the stability boundary from the stable to unstable zones. The λ that crosses the boundary is an instability with frequency ω/2π = γϖ/2π, where λ = 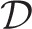
(ϖ) on the boundary.

We now describe some general properties of the stability boundary for structural brain networks with time delays and non-zero dendritic time constants. In the following sections we investigate particular cases. Firstly, 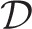 ≠ 0 for all real −∞ < ϖ < ∞, thus λ = 0 does not lie on the stability boundary. If λ = 0 is substituted into Equation (11) then

(12) $${\left(1-i\varpi \right)}^{2}=0,{\left(1-i\varpi \gamma /\alpha \right)}^{2}=0,\text{or }{\left(1-i\varpi \gamma /\beta \right)}^{2}=0,$$

for complex ϖ. The only solutions to Equation (12) are ϖ = −*i*, ϖ = −*i*α/γ, or ϖ = −*i*β/γ giving the dispersion solution ω = −*i*γ, ω = −*i*α, and ω = −*i*β. These solutions all lie in the lower half plane for α, β, γ > 0 and hence λ = 0 is a stable eigenvalue. If ω = ϖ = 0 then 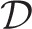
(ϖ) = 1, since *L*(0) = 1, and λ = 1 lies on the stability boundary. This implies that if λ = 1 is in Sp(**G**) then the network has a zero frequency marginally stable dispersion solution. Furthermore, if we consider

(13) $$|\lambda |=|L(\varpi ){|}^{-1}|e^{-i\varpi \gamma \tau }||1-i\varpi {|}^{2},$$

(14) $$=|1-i\varpi \gamma /\alpha ||1-i\varpi \gamma /\beta ||e^{-i\varpi \gamma \tau }||1-i\varpi {|}^{2},$$

then |λ| < 1 implies at least one of the factors on the right of Equation (14) is less than 1. Since α, β, γ, and τ are all positive, this condition can only be satisfied if Imω < 0. Therefore, the unit disk is always contained in the stability zone and if all the eigenvalues of a gain matrix lie in the unit disk the network is stable independent of α, β, γ, and τ.

#### Stability of excitatory networks

If the all the connections in a structural brain network are excitatory then *G*_*ab*_ ≥ 0 and **G** is a non-negative matrix. The Perron–Frobenius theorem (Horn and Johnson, 1985; Cvetković et al., 1995) then implies that **G** has a real eigenvalue λ_*p*_ such that |λ_*i*_| ≤ λ_*p*_ for all λ_*i*_ in Sp(**G**). Therefore, an excitatory brain network is stable if and only if λ_*p*_ < 1; i.e., all the eigenvalues are in the unit disk. This stability criteria follows from the discussion at the end of the previous section and the fact that if λ_*p*_ = 1 then ω = 0 is a solution to Equation (11). Since 0 lies on the stability boundary this implies if λ_*p*_ ≥ 1 then Imω ≥ 0. This means the stability of an excitatory brain network is independent of α, β, γ, and τ (as described in the previous section). In general, structural brain networks have inhibitory connections and the Perron–Frobenius theorem does not apply. This implies the presence of inhibitory connections allows the stability zone to extend beyond the unit disk.

### Impact of time delays and dendritic time constants on network stability

#### Stability of networks with no time delays and instantaneous rise and decay times

For networks with no time delays and instantaneous dendritic rise and decay times (i.e., 1/α = 1/β = τ = 0) 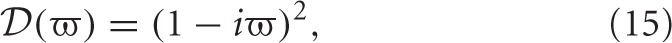 and hence, for each λ in Sp(**G**) there are two dispersion solutions given by

(16) $$\omega =\gamma \varpi =-\gamma (i\pm i\sqrt{\lambda }).$$

Taking the imaginary part of Equation (16) we obtain

(17) $$\text{Im}\omega =-\gamma (1\pm \text{Re}\sqrt{\lambda }).$$

The stability condition Imω < 0 implies that −γ(1 ± Re $\sqrt{\lambda }$) or Re$\sqrt{\lambda }$ ≤ 1 since γ > 0. Thus the stability of a network with 1/α = 1/β = τ = 0 is independent of γ and all λ must satisfy Re$\sqrt{\lambda }$ ≤ 1 or alternatively Reλ + |λ| ≤ 2 [since (Re$\sqrt{\lambda }$)^2^ equals (Reλ + |λ|)/2, with equality corresponding to the stability boundary].

**Figure 1:**
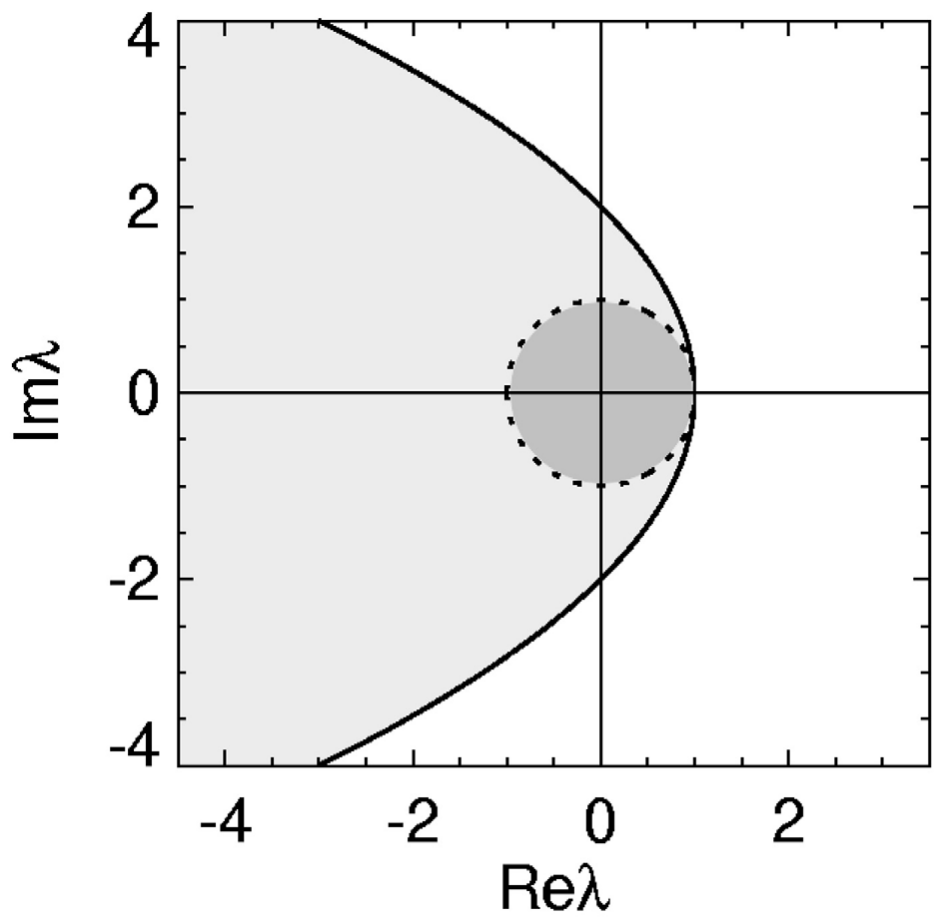
**Stability zone for a brain network with 1/α = 1/β = τ = 0.** The gray zone is where all the eigenvalues λ of the brain network must lie for the network to be stable. The dark region within this zone is the unit disk.

If λ_*r*_ = Reλ and λ_*i*_ = Imλ then the stability zone is a parabolic zone in the complex plane given by

(18) $$\lambda_{i}^{2}\leq 4-4\lambda_{r}$$

with equality giving the stability boundary. The axis of the parabolic boundary is along the real axis with a turning point at (λ_*r*_, λ_*i*_) = (1,0) and imaginary axis intercepts at λ_*i*_ = ± 2. This stability zone is the light gray zone in Figure 1 and is the stability region described previously in (Gray and Robinson, 2008, 2009a,b).

#### Stability of networks with equal time delays

We now consider the effect of time delays on the stability of networks with 1/α = 1/β = 0. In this section we determine how the addition of a time delay to structural brain networks modifies the parabolic stability zone described by Equation (18) and describe the characteristics of the stability boundary. As noted previously, under our assumptions any self-connection in a brain network has the same time delay as connections between populations.

In this case *L*(ω) = 1, τ ≠ 0, and 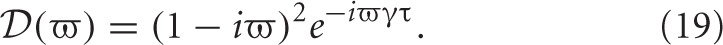


**Figure 2:**
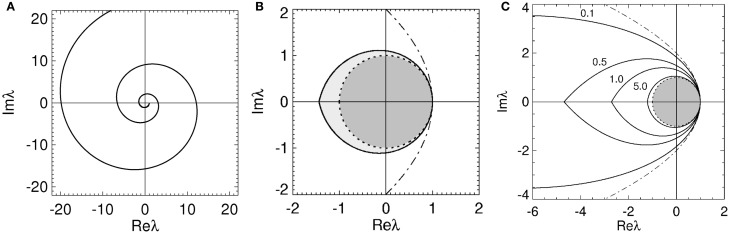
**Stability boundaries and stability zones for networks with 1/α = 1/β = 0.** The curve 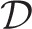
(ϖ) for ϖ ≥ 0 **(A)** and corresponding stability boundary **(B)** for networks with 1/α = 1/β = 0 and γτ = 3.0. The shaded zone in **(B)** is the stability zone for a network and the dark region within this zone is the unit disk. The dot-dashed line is the boundary for 1/α = 1/β = τ = 0. **(C)** Stability boundaries for brain networks with 1/α = 1/β = 0 and γτ = 0.1, 0.5, 1.0, and 5.0. The values of γτ are written slightly above and to the left of the corresponding boundary. The dark gray region within this zone is the unit disk.

Due to the exponential in Equation (19), λ − 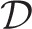
(ϖ) = 0 has an infinite number of solutions for each eigenvalue of the gain matrix. If λ = Reλ + *i*Imλ = λ_*r*_ + *i*λ_*i*_ then λ = 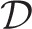
(ϖ) (for real ϖ) implies 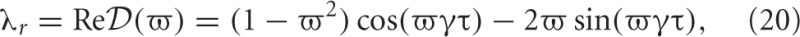 and 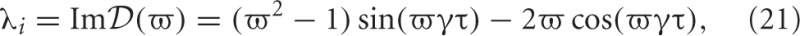 these equations can be combined giving

(22) $$\lambda_{i}=-\tan(\varpi \gamma \tau )[\lambda_{r}+2\varpi \text{cosec}(\varpi \gamma \tau )],$$

which gives the stability boundary in the complex plane. For ϖ ≥ 0, Equation (22) describes a spiral curve in the complex plane starting at the point (λ_*r*_, λ_*i*_) = (1, 0), centered on the origin, and spiraling in a clockwise direction as ϖ increases, as seen in Figure 2A. For ϖ < 0, Equation (22) describes a similar counterclockwise spiral curve, corresponding to a reflection of the curve in Figure 2A about the imaginary axis.

Using Equations (20–22) we now describe the characteristics of the stability boundary and the resulting stability zone. The 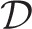
(ϖ) curve crosses the real axis when λ_*i*_ = 0. Substituting λ_*i*_ = 0 into Equation (21) and (22) gives

(23) $$(\varpi^{2}-1)\text{sin}(\varpi \gamma \tau )=2\varpi \text{cos}(\varpi \gamma \tau ),$$

and

(24) $$\lambda_{r}=-2\varpi \text{cosec}(\varpi \gamma \tau ),$$

respectively. The values of ϖ for which λ_*i*_ = 0 can be obtained by solving Equation (23) numerically. Note that Equation (24) is only valid if ϖγτ ≠ ±*m*π/2 for integers *m*. Due to the periodicity of the sine and cosine functions there is an infinite number of ϖ that satisfy these equations.

Thus the stability boundary consists of two spiral curves produced by bending the arms of the parabola described by Equation (18) inwards. These spiral curves intersect an infinite number of times on the real axis enclosing larger and larger regions of the complex plane as |ϖγτ| increases. The intersection of all these enclosed regions, corresponding to the innermost zone, represents the stability zone for the network. The reason eigenvalues outside this innermost zone are instabilities is because they correspond to eigenvalues outside the stability zone described by Equation (18) when transformed through multiplication by *e*^*i*ϖγτ^ (which removes the effect of the time delay).

We define the smallest ω = γϖ > 0 that gives λ_*i*_ = 0 the *critical* ω value. This critical value is denoted by ω_*c*_ with the corresponding ϖ and λ_*r*_ denoted ϖ_*c*_ and λ^*c*^_*r*_, respectively. The stability zone is defined by 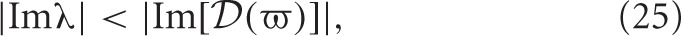 for 0 ≤ ϖ ≤ ϖ_*c*_. This zone has a boundary defined by Equation (19) for −ϖ_*c*_ ≤ ϖ ≤ ϖ_*c*_. The real axis intercepts of the stability boundary are given by λ_*r*_ = 1 and λ_*r*_ = λ^*c*^_*r*_ = −2ϖ_*c*_cosec(ϖ_*c*_γτ). As seen in Figure 2B the stability zone has a teardrop shape containing the unit disc. Note that Equation (25) also defines the stability zone in Figure 1 with ϖ_*c*_ = ∞. As γτ → 0 then λ_*r*_ → (1 − ϖ^2^) in Equation (20), λ_*i*_ → −2ϖ in Equation (21), and the stability boundary converges to the parabola λ^2^_*i*_ = 4 − 4λ_*r*_, as expected.

The effect of increasing τ on the stability boundary is shown in Figure 2C. As γτ increases, the stability boundary converges to the unit circle with the stability zone converging to the unit disk, shaded dark gray. The values of ϖ_*c*_ in Figure 2C for the four values of γτ shown are 3.0 (γτ = 0.1), 1.92 (γτ = 0.5), 1.31 (γτ = 1.0), and 0.46 (γτ = 5.0), respectively. The corresponding λ^*c*^_*r*_ are −9.4 (intersection not seen), −4.6, −2.7, and −1.2. For γ = 100 s^−1^ and τ = 0.01 s (the nominal values in Table 1) ω_*c*_/2π ≈ 30 Hz.

Overall, the presence of time delays bends the parabola described by Equation (18) inward, forming a teardrop-shaped stability zone containing the unit disk. As τ → ∞ the stability boundary wraps around the unit circle an infinite number of times and the stability zone converges to the unit disk, restricting the critical frequency.

#### Stability of networks with non-zero dendritic rise and decay time constants and no time delays

The teardrop shaped zone and the time-delay effects on stability described in the previous section produce similar results to those seen in other studies (Marcus and Westervelt, 1989; Jirsa and Ding, 2004; Feng et al., 2006). However, our model also incorporates dendritic rise and decay time constants. In the next two sections we describe the stability of structural brain networks with non-zero dendritic time constants.

We first investigate brain networks with dendritic time constants and no propagation time delays. In this case τ = 0, α ≠ 0, and β ≠ 0. Hence *L*(ω) ≠ 1 and 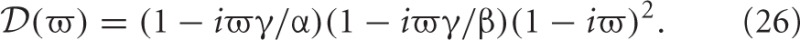


From Equation (26) the stability boundary is given by

(27) $$\lambda_{r}=1-[1+2\gamma (1/\alpha +1/\beta )+\gamma^{2}/(\alpha \beta )]\varpi^{2}+\gamma^{2}/(\alpha \beta )\varpi^{4},$$

and

(28) $$\lambda_{i}=-[2+\gamma (1/\alpha +1/\beta )]\varpi +[\gamma (1/\alpha +1/\beta )+2\gamma^{2}/(\alpha \beta )]\varpi^{3}.$$

where λ_*r*_ = Reλ, Imλ = λ_*i*_, and ϖ is real. In this case λ − 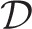
(ϖ) = 0 only has a finite number of solutions since 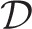
(ϖ) is a polynomial of degree four. From Equation (28) the values of ϖ_*c*_ and ω_*c*_ are given by

(29) $$\varpi_{c}^{2}=\frac{2\alpha \beta +\gamma (\alpha +\beta )}{2\gamma^{2}+\gamma (\alpha +\beta )},$$

and

(30) $$\omega_{c}=\sqrt{\frac{2\alpha \beta \gamma +\gamma^{2}(\alpha +\beta )}{\alpha +\beta +2\gamma }},$$

respectively. An equation similar to Equation (30) was previously derived to describe gamma resonances produced by a similar mechanism (Robinson, 2005). If αβ < γ^2^ then Equation (30) implies ω_*c*_ < γ.

**Figure 3:**
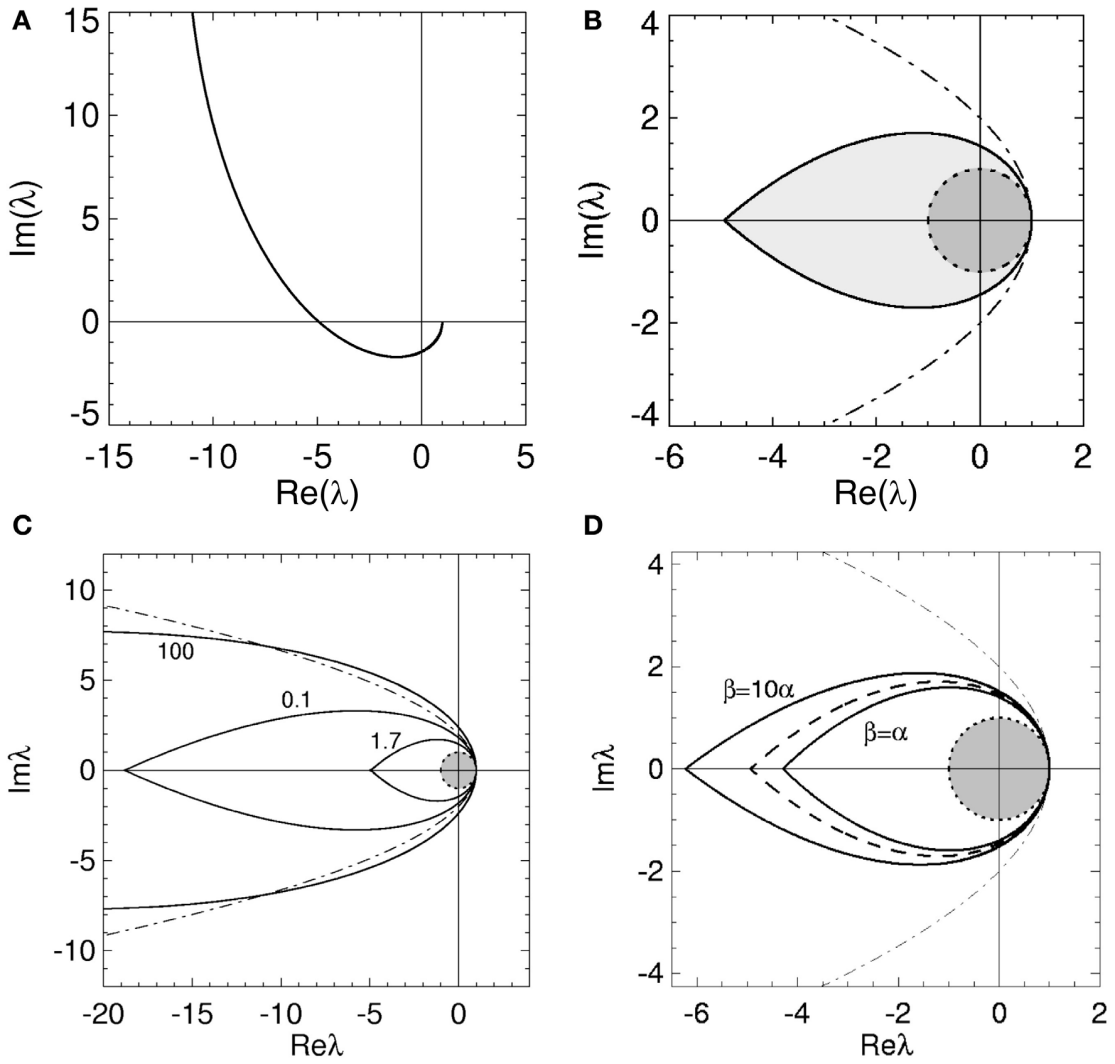
**Stability boundaries and stability zones for networks with non-zero dendritic rise and decay time constants and no time delays.** The curve 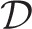
(ϖ) for real ϖ ≥ 0 **(A)** and corresponding stability zone and boundary **(B)** for networks with α = 60 s^−1^, β = 240 s^−1^ and γ = 100 s^−1^ and τ = 0 s. The stability zone is shaded gray and the dark region within this zone is the unit disk. The dot-dashed line is the boundary for 1/α = 1/β = τ = 0. **(C)** Stability boundaries for β = 4α and γ/α = 100, 0.1, and 1.7. The values of γ/α are written next to their corresponding boundary. **(D)** Stability boundaries for γ/α = 1.7 and β/α = 1, 4 (dashed curve), and 10. The dot-dashed line in **(C,D)** is the boundary for 1/α = 1/β = τ = 0 and the dark gray region in **(C,D)** is the unit disk.

Unlike the case for time delays in the previous section, the 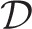
(ϖ) curve only crosses the real axis once for real ϖ > 0; an example of such a 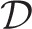
(ϖ) curve is shown in Figure 3A. However, the region enclosed by 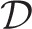
(ϖ) for −ϖ_*c*_ ≤ ϖ ≤ ϖ_*c*_ is again the stability zone and defined by Equation (25). The corresponding stability zone for the 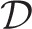 curve in Figure 3A is shown in Figure 3B.

The stability zone in Figure 3B contains the unit disk and has a similar teardrop shape to the zone in Figure 2B with the arms of the parabola given by Equation (18) bent inward. In terms of stability, this implies non-zero 1/α and 1/β have similar effects to a propagation time delay. This is consistent with previous work on the corticothalamic model and highlights the low-pass filter effect of *L*(ω) (Robinson et al., 1997, 2001a,b; Rennie et al., 2002). The effective time delay resulting from γ/α and γ/β can be obtained by solving

(31) $$e^{-i\varpi_{c}\gamma \tau }=(1-i\varpi_{c}\gamma /\alpha )(1-i\varpi_{c}\gamma /\beta ),$$

for τ.

**Figure 4:**
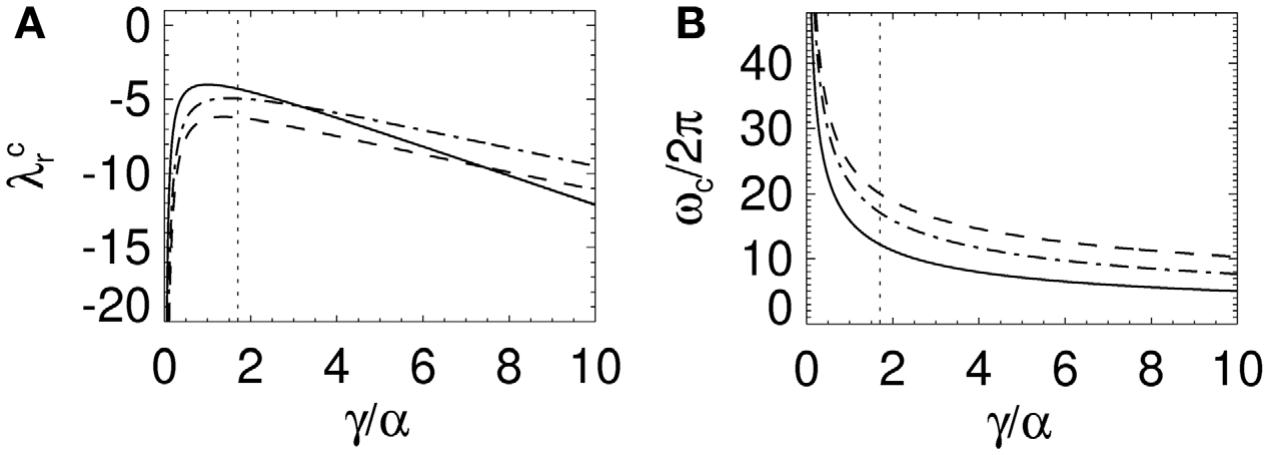
**Change in stability boundary real axis intercept and critical frequency as a function of γ/α. (A)** λ^*c*^_*r*_ and **(B)** ω_*c*_ as a function of γ/α for γ τ = 0 and β/α = 1 (solid lines), 4 (dotted lines), and 10 (dot-dashed lines). In **(B)** ω_*c*_ is determined with γ = 100 s^−1^. The dotted lines represent the nominal γ/α from Table 1.

In the remainder of this section we explore the effect of varying γ, α, and β on the stability zone. We illustrate these effects by setting β/α to a positive constant. Firstly, for fixed β/α and large γ/α, Equations (27, 29) imply

(32) $$\lambda_{r}\approx 1+{\left(\gamma /\alpha \right)}^{2}\varpi^{2}(\varpi^{2}-1),$$

and

(33) $$\varpi_{c}^{2}\approx \alpha /\gamma ,$$

respectively. From Equation (33), ω_*c*_ ≈ 0 for large γ/α and substituting Equation (33) into (32) shows that λ^*c*^_*r*_ → −∞ as γ/α → ∞. These results are illustrated in Figures 3C, 4.

The change in the stability zone for fixed β/α and varying γ/α is shown in Figure 3C. All the stability zones contain the unit circle, and for γ/α = 0.1 and 1.7 the stability zone is contained within the parabolic zone defined by Equation (18). However, the zone for γ/α = 100 has expanded so that its boundary intersects the parabolic boundary at λ_*r*_ ≈ −11. The ϖ_*c*_ in Figure 3C for γ/α = 100, 0.1, and 1.7 are 0.15, 4.0, and 1.08, respectively. The corresponding λ^*c*^_*r*_ are −66 (intersection not seen), −19, and −4.9. For γ/α = 1.7, the nominal value from Table 1, the critical frequency is ω_*c*_/2π ≈ 17 Hz.

The effect of changing β/α on the stability zone, while γ/α remains fixed, is shown in Figure 3D. The ϖ_*c*_ (λ^*c*^_*r*_) in Figure 3D for β/α = 1 and β/α = 10 are 0.77 (λ^*c*^_*r*_ = −4.3) and 1.26 (λ^*c*^_*r*_ = −6.2), respectively. Note that if β/α < 1, then the values of α and β can be swapped and the results in Figure 3 are reproduced. This is because exchanging γ/α and γ/β in Equation (4) has no effect on the dynamics and stability of a network. This implies the smallest stability zone with the minimum λ^*c*^_*r*_ occurs when α = β. Note that experimental measurements of dendritic time constants in the brain give β/α ≈ 4–10 (Robinson et al., 2003a, 2004), the upper range in Table 1, and hence, a larger stability zone for brain activity.

Figure 3 suggests that as β/α increases the stability zone expands in a similar way to decreasing τ. However, as τ → 0 the stability zone converges to the parabolic zone (Equation 18), this is not the case for γ/β → ∞ and fixed γ/α. If γ/α is fixed to a positive constant and γ/β » γ/α then, from Equation (29), ϖ^2^_*c*_ ≲ 1 and therefore

(34) $$\lambda_{r}\approx 1-\gamma \varpi^{2}/\beta ,$$

and

(35) $$\lambda_{i}\approx -\gamma \varpi /\beta .$$

When λ_*r*_ = 0, Equation (34) implies $\varpi \approx \sqrt{\beta /\gamma }$ and $\lambda_{i}\approx -\sqrt{\gamma /\beta }$. Therefore as γ/β → ∞, the imaginary axis intercepts converge to ± ∞ and the stability zone expands to cover the entire region of the complex plane defined by Reλ < 1. Note that in this case, even though the eigenvalues can lie anywhere to the left of Reλ = 1, the dispersion solutions have an angular frequency ω < γ. These results explain the intersection of the stability boundary with the parabolic boundary in Figure 3C.

With Figure 3C these convergence results suggest that as γ/α increases from 0, the stability zone contracts toward the unit circle, and then expands again. Figure 4 shows the values of λ^*c*^_*r*_ and ω_*c*_ as a function of γ/α and β/α. As γ/α increases from 0, λ^*c*^_*r*_ rapidly increases from −∞ to a maximum value and then slowly decreases back to −∞; this decrease is greatest for the β/α = 1 curve which intersects the other two curves in Figure 4A. The maximum turning point for λ^*c*^_*r*_ occurs when γ ≈ α for each β/α, with maximum λ^*c*^_*r*_ decreasing as β/α increases. When β/α = 4 (which is the nominal value in Table 1), λ^*c*^_*r*_ is approximately constant for 1 ≲ γ/α ≲ 3, with a maximum at γ/α ≈ 1.7, the nominal value. The corresponding ω_*c*_ curves in Figure 4B all show similar monotonic decreases as γ/α increases. The curves do not intersect or have a turning point as in Figure 4A. This decrease from ∞ is initially very rapid, before gradually decreasing to 0 as γ/α → ∞. This change approximately occurs at the nominal γ/α value in Table 1, where ω_*c*_/2π ≲ 20 Hz for each β/α. Note that when β/α is a fixed constant, Equation (33) shows that ω_*c*_ → 0. Figure 4B also shows that increasing γ/α and decreasing γ/β results in a decreased value for ω_*c*_.

In this section we have shown that physiologically realistic dendritic time constants have an effect on network stability similar to that of propagation time delays restricting, the critical frequency and the stability zone to a teardrop-shaped zone in the complex plane. However, unlike τ, for particular values of γ/α and γ/β the stability zone can expand to enclose an area outside the parabolic region described by Equation (18).

#### Stability of networks with time delays and non-zero dendritic time constants

The effect of having both time delays and non-zero dendritic time constants on stability is now described. In this case each of the parameters γ, 1/α, 1/β, and τ are non-zero and 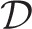 is given by Equation (11). As in the previous section, the stability boundary for these networks is defined by λ_*r*_ = Re
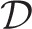
(ϖ) and λ_*i*_ = Im
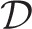
(ϖ) for −∞ < ϖ < ∞. Analysis of these equations gives the properties of the stability boundary and zone. However, the effects on stability of having equal time delays and dendritic time constants are easily understood qualitatively as a combination of our previous results. Beginning with a network that has γ/α = γ/β = γτ = 0 and a parabolic stability zone, given by Equation (18), adding a time delay τ contracts the stability zone toward the unit circle, by “pulling in” the parabolic boundary, forming a teardrop-shaped zone within the original parabolic region. Adding dendritic rise and decay constants then, depending on their value, expands or contracts this stability zone. In all cases the stability zone is defined by 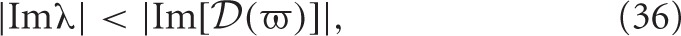 for 0 ≤ ϖ ≤ ϖ_*c*_. Note that for large γτ, γ/α, and γ/β the contraction caused by γτ is greater than the expansion effects due to γ/α and γ/β because of the exponential in Equation (11).

**Figure 5:**
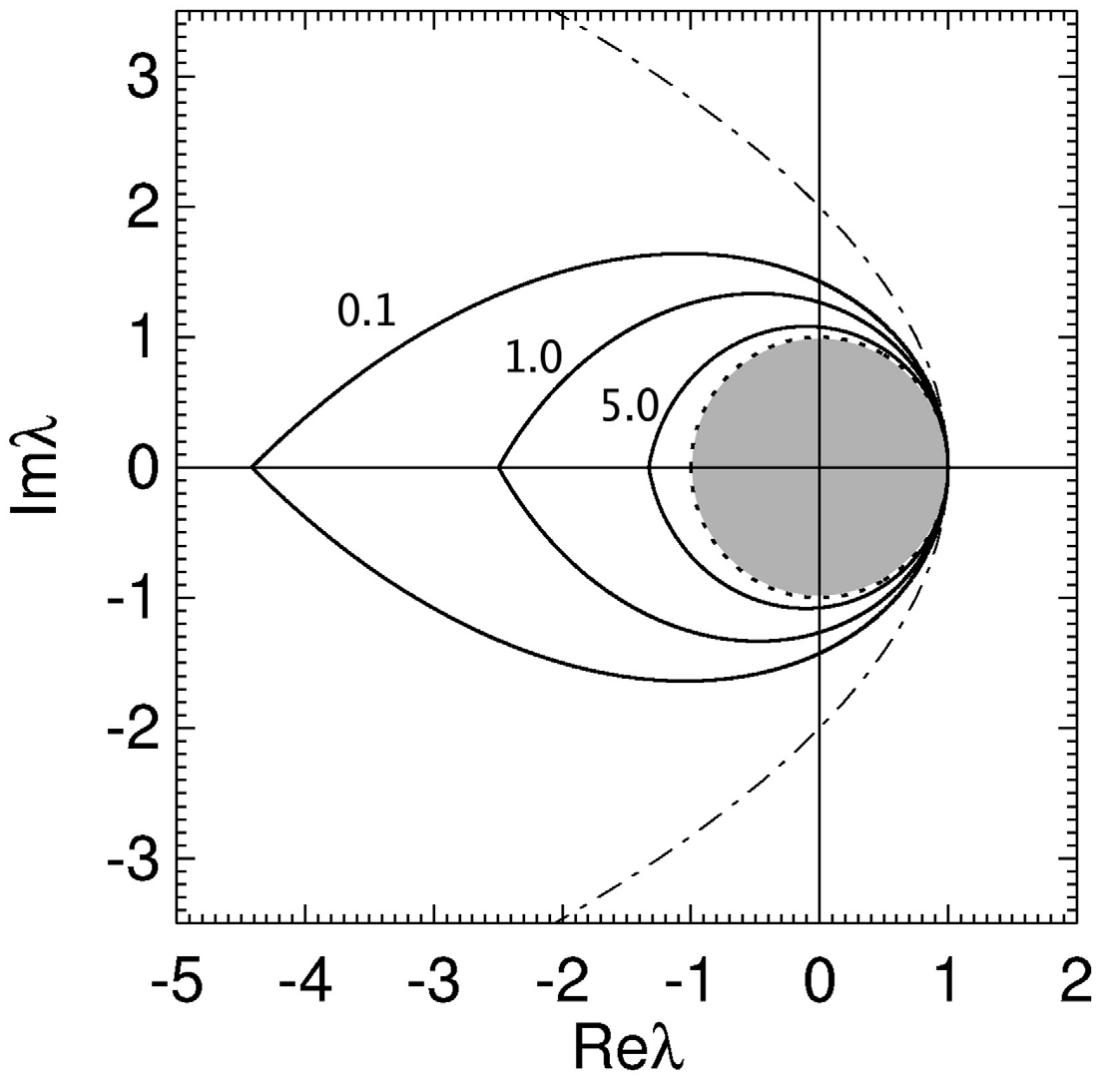
**Stability boundaries for brain networks with γ/α = 1.7, β/α = 4 and γτ = 0.1, 1.0, and 5.0.** The values of γτ are written slightly to the left of their corresponding boundary. The gray region is the unit disk.

In Figure 5 the stability zone of a brain network with plausible time delays and dendritic time constants (from Table 1) is shown. This shows that for realistic parameter values brain networks have a teardrop-shaped stability zone completely within the parabolic zone (Equation 18). For large realistic τ the stability zone is only slightly larger than the unit disc. The ϖ_*c*_ in Figure 5 for increasing γτ are 1.02, 0.72, and 0.35, respectively. The corresponding critical frequencies ω_*c*_/2π for γ = 100 are then 16, 11.3, and 5.5 Hz, respectively.

### Stability and dispersion solutions of randomly connected networks

We now investigate the stability and dispersion solutions of the randomly connected structural brain networks defined in the “Methods” section. For these networks we fix the model parameters γ, α, β, and τ to their nominal values in Table 1. The stability and dispersion solutions of this networks are determined from Sp(**G**). If all the eigenvalues λ in Sp(**G**) satisfy Equation (25) then the network is stable. The corresponding dispersion solutions are obtained numerically by solving Equation (10) for each λ using CROOT (Botten et al., 1983) (as described in “Methods”).

The spectrum of a RENs consists of one eigenvalue at *np*μ_*e*_ with the other *n* − 1 eigenvalues uniformly distributed in a disc of radius $\mu_{e}\sqrt{np(1-p)}<np\mu_{e}$. The spectrum of RCNs and RPNs with maximum μ_*e*_ and μ_*i*_ allowed by stability is distributed within the unit disk, see (Gray and Robinson, 2009a), with multiple eigenvalues near the stability boundary. Therefore, stability constrains the spectrum of random brain networks to the unit disk and the stability of random brain networks is independent of the γ, α, β, and τ. However, the frequencies of the dispersion solutions do depend on the model parameters.

**Figure 6:**
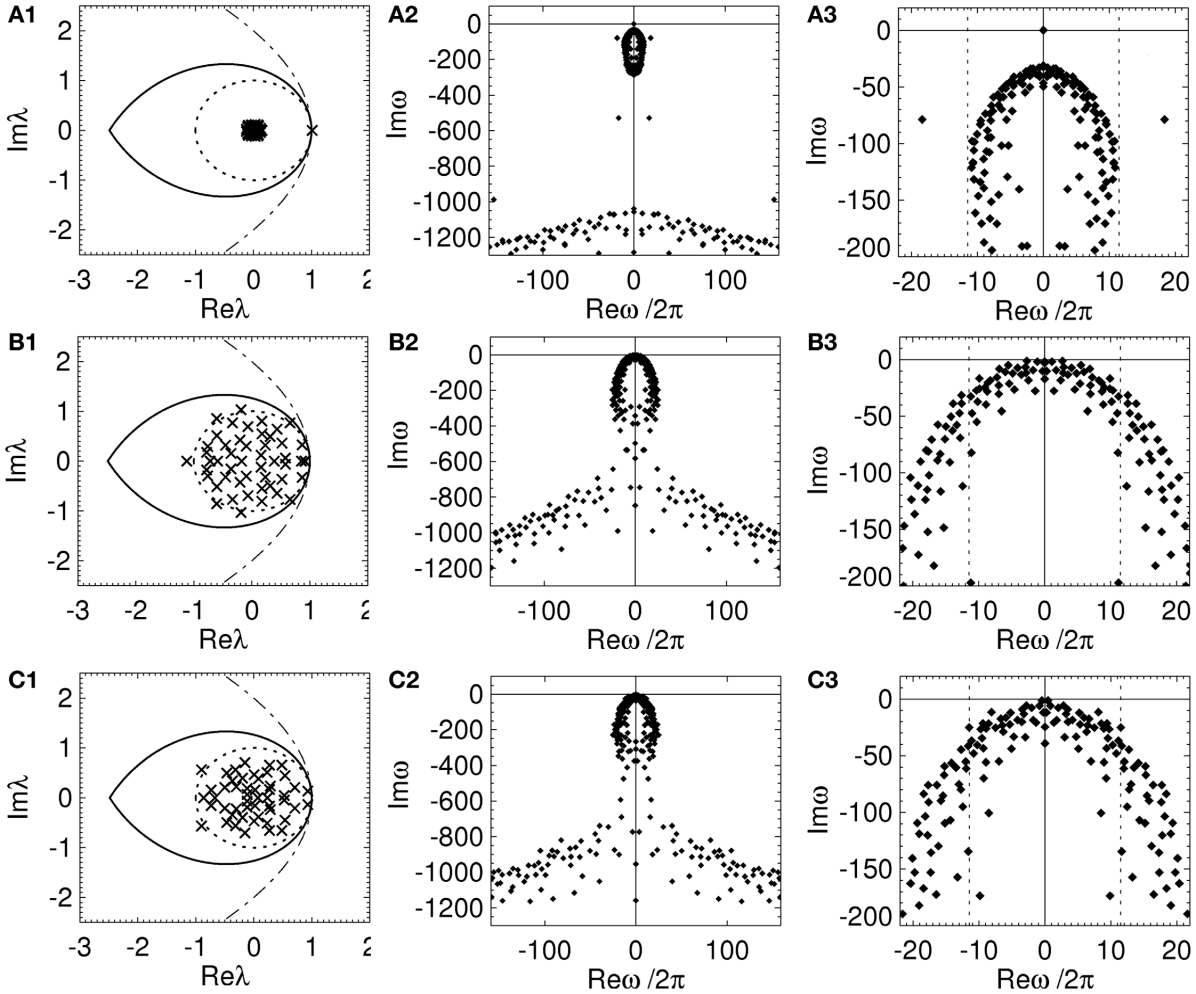
**Spectrum and dispersion solutions for a REN, a RCN, and a RPN.** Each network has *n* = 50, *p* = 0.5, and the nominal parameters in Table 1, other parameters are set so that the network is close to marginal stability. The left column is the spectrum (crosses), the middle column shows the dispersion solutions (diamonds), and the right column shows an expanded view of the dispersion solutions near the imaginary axis with dotted lines representing the critical frequencies ± ω_*c*_/2π. **(A)** REN with μ_*e*_ = 0.04, **(B)** RCN with *p*_*i*_ = 0.5, μ_*e*_ = −μ_*i*_ = 0.2, σ_*e*_ = σ_*i*_ = 0, and **(C)** RPN with *p*_*i*_ = 0.5, μ_*e*_ = −μ_*i*_ = 0.22, σ_*e*_ = σ_*i*_ = 0.

In Figure 6 the spectrum and dispersion solutions for a REN, RCN, and RPN with the nominal model parameters are shown. The parameters of each network are set so that the networks are marginally stable and μ_*e*_ and |μ_*i*_| are as large as possible while maintaining stability. Note the larger values of μ_*e*_ and |μ_*i*_| for the RPN, compared to the RCN. This highlights that RPNs can have larger μ_*e*_ and |μ_*i*_|, and hence be more responsive, before becoming almost certainly unstable as shown in (Gray and Robinson, 2009a). This suggests stability may have an effect on the arrangement of inhibitory and excitatory neurons and their physiology in structural brain networks.

Each network has an infinite number of dispersion solutions because τ ≠ 0. The second column of Figure 6 shows the dispersion spectrum is symmetrically placed around the real axis. Each network has a qualitatively similar dispersion spectrum, with a finite cluster of solutions near the origin and a broad “arrowhead” of solutions for Imω ≲ 800 s^−1^; this arrowhead has an infinite number of solutions with Imω decreasing as |Reω| increases. The third column shows the dispersion solutions near the imaginary axis. In Figure 6A3 one solution, ω = ω_1_, lies on the origin, separate from the other dispersion solutions. This solution corresponds to the eigenvalue at λ ≈ 1 that is separated from the rest of the spectrum in Figure 6A1. This implies the dynamics of an REN will be dominated by a zero-frequency mode. Since the rest of the dispersion solutions have Imω « 0 all other modes rapidly decay to zero amplitude. However, the RCN and RPN in Figure 6 have a very similar dispersion spectrum with multiple dispersion solutions near the imaginary axis. The solutions closest to the imaginary axis have small frequencies ≲ 5 Hz. This shows that the presence of inhibitory connections allows random networks to have multiple marginally stable low frequency modes (Gray and Robinson, 2008, 2009a,b).

## Discussion

We increased the physiological realism of a structural brain network model we studied previously in (Gray and Robinson, 2006, 2008, 2009a,b; Robinson et al., 2009) by allowing the network to have equal time delays τ for propagation between neuronal populations and non-zero dendritic rise 1/β and decay 1/α time constants. Under these assumptions the stability of an arbitrarily connected network of neural populations is determined by the network's gain matrix. The addition of time delays changed the stability zone in the complex plane from a parabolic region to a teardrop-shaped zone, dependent on α, β, τ, and the temporal damping rate γ. Our results are similar to previous work investigating the effect of time delays on stability of electrical activity within spatially continuous networks of neural tissue (Marcus and Westervelt, 1989; Jirsa and Ding, 2004; Feng et al., 2006; Qubbaj and Jirsa, 2007, 2009; Jirsa, 2009). This previous work has generally used integro-differential neural field equations with connectivity within a neural mass described by homogeneous or heterogeneous kernels. While in principle this work could be applied to large-scale connection topologies of discrete neural masses, as we have investigated here, this has generally not been done as it is difficult to incorporate arbitrary connectivity patterns (Qubbaj and Jirsa, 2009). In this work we have investigated the temporal dynamics of the overall electrical activity of arbitrarily connected structural brain networks, ignoring the spatial spread and propagation of electrical activity within individual neuronal populations.

In terms of stability the effect of non-zero dendritic time constants is similar to a time delay, further suggesting that dendrites act as a low-pass filter on synaptic inputs (Robinson et al., 1997, 2001a,b; Rennie et al., 2002). However, dendritic time constants can change the shape of the stability zone even allowing it to expand and enclose areas outside the parabolic stability region for networks with zero time delays and instantaneous dendritic rise and decay times. For all values of γ, α, β, and τ the stability zone contains the unit disk. This result implies that the stability criteria originally derived by May (1972, 1974) (that a network is stable if all its eigenvalues lie in the unit disk) is a sufficient condition for the stability of structural brain networks.

We also explored the dispersion solutions and frequencies of structural brain networks. If an initially stable brain network becomes unstable through a change in its connection gains, then at least one eigenvalue moves across the stability boundary and the network has an instability at a frequency given by the eigenvalue's corresponding dispersion solution. For networks with time delays and non-zero dendritic time constants there is a maximum frequency, the critical frequency ω_*c*_/2π, at which initially stable networks will become unstable. For example, if all the gain matrix eigenvalues of a networks are initially inside the stability zone but then move across the stability boundary (e.g., due to changes in connection gains) then the frequency of these instabilities will be less than the critical frequency.

Measurements of brain activity (Stam et al., 1999; Robinson et al., 2001b; Breakspear, 2002; Breakspear et al., 2003) suggest the brain operates near marginal stability allowing the brain to have rich dynamics and a wide range of complex behavior. A network near marginal stability has eigenvalues near the stability boundary with corresponding modes that are the slowest to decay back to the steady state dominating the network's dynamics. These modes have a frequency less than the critical frequency. Using physiologically plausible parameter values in our structural network model (see Table 1) we would expect the electrical dynamics to be dominated by frequencies ≲100 Hz. When the nominal parameter values of α, β, γ, and τ are used the critical frequency is approximately 10 Hz and decreases as γτ increases (see Figures 4, 6).

For the randomly connected structural brain networks we investigated previously (Gray and Robinson, 2006, 2008, 2009a,b) the spectrum of the gain matrix is almost certainly contained in a disk centered on the origin with a radius dependent on the network's architecture and the average values of its excitatory and inhibitory gains. Thus, the stability zone of these networks is the unit disk and their stability is independent of time delays and dendritic time constants. Therefore, the results of that work remain valid in the more general case studied here. However, for the critical frequency is dependent on dendritic time constants, temporal damping rate, and time delays. We showed marginally stable randomly connected networks with inhibitory connections have multiple marginally stable low frequency dispersion solutions.

The primary goal of this and our previous work on structural brain networks is to understand how stability potential constrains the structure physiology of networks. For the randomly connected networks studied in (Gray and Robinson, 2006, 2008, 2009a,b), we have shown that time delays and non-zero dendritic time constants have minimal effect on their stability. One network type whose stability could be affected by these physiological properties is networks with inhibitory self-connections. The spectrum of these networks, even if they are randomly connected, is no longer restricted to a disc by stability but can have eigenvalues distributed within the teardrop-shaped region. Such networks could have marginally stable modes with frequencies (up to the critical frequency) in the alpha, beta, and gamma ranges. This will be explored in future work.

### Likely effects of distributed or varying parameters on stability

Assuming structural brain networks have equal γ, α, and β for each neural population and equal τ for each connection is unrealistic. Different neuronal populations in the brain have different parameter values; for example, excitatory cortical neurons have γ ≈ 100 s^−1^ while for inhibitory cortical neurons γ ≈ 10^3^–10^4^ s^−1^ (Robinson et al., 2004). Also, the time delay in real cortical networks is expected to vary from τ = 0 for self-connections to a large value for areas physically far apart. A realistic model of a structural brain network would therefore allow the model parameters γ, α, β, and τ to vary across neural populations. This variation could possibly be represented as a distribution.

The effect of distributed time delays on network stability has been studied using general models for network activity (Yi and Tan, 2002; Atay, 2003; Jirsa and Ding, 2004; Feng et al., 2006). In (Feng et al., 2006) and (Jirsa and Ding, 2004) networks with a distributed time delay with mean τ were shown to have a stability zone that contained the stability zone of networks with an constant delay equal to τ. These results are applicable to the brain networks studied here, since our model without dendritic time constants can be described as a specific case of the model studied in (Jirsa and Ding, 2004; Feng et al., 2006). This implies brain networks with distributed delay are more stable than networks with equal time delays; in the sense that a stable network with a distribution of delays could be unstable if its delays were replaced with a constant delay equal to the distribution mean. Hence, the equal time delay case is the least stable case and yields a bound on the stability of a structural brain network.

Here we have shown that plausible dendritic time constants have similar effects on stability as a time delay. This suggests that similar results to those found in (Jirsa and Ding, 2004; Feng et al., 2006) will likely be observed for distributed α and β. Also, we have shown that γ only affects the dispersion frequencies of a network, not its stability. A distributed γ is therefore expected to have no effect on the stability, only on the dispersion frequencies but this needs to be confirmed numerically.

Given the previous results investigating distributed parameter values we argue our results are still informative. But the exact effect of distributed γ, α, and β on structural brain network dynamics needs to be determined, particularly to understand the critical frequency and the dynamics of marginally stable modes in a networks electrical activity. To fully understand the stability and dynamics of structural brain networks with varying time delays and dendritic time constants requires a numerical approach. This will be investigated using CROOT (Botten et al., 1983) in future work.

## Conclusions

We investigated the stability of discrete networks of neuronal populations using a simplified physiologically-based mean-field model of brain electrical activity. Incorporating time delays and non-zero dendritic time constants affects the stability of arbitrarily connected structural brain networks by constraining the eigenvalues of the gain matrix to a teardrop-shaped region in the complex plane. The stability of randomly connected networks of excitatory and inhibitory neuronal populations is unaffected by time delays and dendritic time constants; as stability constrains the gain matrix eigenvalues to the unit circle. However, the dispersion frequencies of instabilities are affected by network physiology. Randomly connected brain networks with the largest average excitatory and inhibitory gains allowed by stability can have multiple marginally stable low-frequency modes. Such networks would be highly responsive and adaptable to external stimuli while remaining stable, and have a wide range of flexible, adaptable, and complex behavior.

### Conflict of interest statement

The authors declare that the research was conducted in the absence of any commercial or financial relationships that could be construed as a potential conflict of interest.
